# MicroPET/CT Imaging of AXL Downregulation by HSP90 Inhibition in Triple-Negative Breast Cancer

**DOI:** 10.1155/2017/1686525

**Published:** 2017-05-14

**Authors:** Wanqin Wang, Jun Zhao, Xiaoxia Wen, Curtis Chun-Jen Lin, Junjie Li, Qian Huang, Yongqiang Yu, Shiaw-Yih Lin, Chun Li

**Affiliations:** ^1^Department of Cancer Systems Imaging, The University of Texas MD Anderson Cancer Center, Houston, TX, USA; ^2^Department of Radiology, The 1st Affiliated Hospital of Anhui Medical University, Hefei, Anhui, China; ^3^Department of Systems Biology, The University of Texas MD Anderson Cancer Center, Houston, TX, USA

## Abstract

AXL receptor tyrosine kinase is overexpressed in a number of solid tumor types including triple-negative breast cancer (TNBC). AXL is considered an important regulator of epithelial-to-mesenchymal transition (EMT) and a potential therapeutic target for TNBC. In this work, we used microPET/CT with ^64^Cu-labeled anti-human AXL antibody (^64^Cu-anti-hAXL) to noninvasively interrogate the degradation of AXL in vivo in response to 17-allylamino-17-demethoxygeldanamycin (17-AAG), a potent inhibitor of HSP90. 17-AAG treatment caused significant decline in AXL expression in orthotopic TNBC MDA-MB-231 tumors, inhibited EMT, and delayed tumor growth in vivo, resulting in significant reduction in tumor uptake of ^64^Cu-anti-hAXL as clearly visualized by microPET/CT. Our data indicate that ^64^Cu-anti-hAXL can be useful for monitoring anti-AXL therapies and for assessing inhibition of HSP90 molecular chaperone using AXL as a molecular surrogate.

## 1. Introduction

The majority of breast cancer fatality stems from disease relapse due to invasion and metastasis [1]. It is thought that the activation of epithelial-to-mesenchymal transition (EMT) in tumor cells is closely correlated with metastasis and treatment failure [2, 3]. Triple-negative breast cancer (TNBC) constitutes 15%–20% of all breast cancers. Treatment of TNBC is a challenge owing to the heterogeneity of the disease and the lack of actionable targets [4, 5]. There is an unmet medical need to identify and validate molecular targets for TNBC.

AXL is a member of the receptor tyrosine kinase TAM subfamily comprising TYRO-3, AXL, and MER. AXL is often activated by its natural ligand GAS6 (growth arrest-specific protein 6). Activated AXL subsequently activates the MAPK/ERK and PI3K/AKT signaling pathways, leading to tumor growth and invasion [6]. AXL is known to be an important regulator of EMT in breast cancer [7], neuroblastoma [8], and non-small-cell lung cancer [9]. Elevated AXL expression is associated with poor outcome in tumors of the lung [10], breast [11], and pancreas [12]. It also correlates with drug resistance [13, 14]. Meyer et al. [15] showed interplay between EGFR and AXL in TNBC cells and a possible role of AXL in the resistance to EGFR inhibitors. Accordingly, AXL downregulation has demonstrated antitumor activity [16–18]. The first-in-class small-molecular-weight AXL inhibitor BGB324 (formerly R428) was brought to clinical trials in 2013 [19]. In a recent study based on 1,789 tyrosine-phosphorylated peptides identified from 969 proteins, AXL was found to be activated in a majority of aggressive TNBC cell lines [20]. Taken together, AXL is a potential therapeutic target for TNBC.

Because of the emerging role of AXL in TNBC and other cancers, it is important to assess whether therapeutic attenuation of AXL expression could be noninvasively imaged and quantified, which would provide valuable information for assessing the response to therapies that modulate AXL expression level. Because small-molecular-weight AXL inhibitor R428 only affected the phosphorylation of AXL but not AXL protein level, we chose the inhibitor of heat shock protein 90 (HSP90), 17-allylamino-17-demethoxygeldanamycin (17-AAG), as a model drug for AXL downregulation. Earlier studies have shown that AXL is one of the client proteins of HSP90 and that AXL level could be downregulated by HSP90 inhibitors [21, 22]. The purpose of this study was to investigate whether microPET/CT with ^64^Cu-labeled anti-AXL antibody could be used for monitoring downregulation of AXL by 17-AAG.

## 2. Experimental Section

### 2.1. Chemistry and Radiochemistry

Anti-hAXL antibody or IgG was mixed with* p*-SCN-Bn-DOTA at a molar ratio of 1 : 50 in a 1 M sodium bicarbonate buffered solution (pH 8.5). The reaction mixtures were incubated at 4°C overnight, and then the DOTA-conjugated antibodies were purified on a PD-10 column (GE Healthcare, Marlborough, MA) using phosphate-buffered saline solution (PBS) as the eluent. For radiolabeling, an aliquot of ^64^CuCl_2_ (2 *µ*L, 74 MBq in 0.1 N HCl) was diluted in 40 *µ*L of 0.1 M sodium acetate buffer (pH 6.0). The resulting solution was then added to an aqueous solution of DOTA-anti-hAXL or DOTA-IgG at a ratio of 18.5–37 MBq ^64^Cu per 25 *µ*g antibody. After incubation at 45°C for 45 min, the reaction mixture containing radiolabeled anti-hAXL and IgG was subjected to instant thin layer chromatography (ITLC) analysis to determine radiolabeling efficiency.

To determine the stability of ^64^Cu-labeled antibodies, ^64^Cu-anti-hAXL or ^64^Cu- IgG was incubated in PBS containing 20% mouse plasma at 37°C for 48 h. The radioactivity at the original spot was recorded as a percentage of the total radioactivity of the ITLC strip. The ^64^Cu-antibody conjugates were purified on PD-10 columns.

### 2.2. In Vitro Cell Binding Assay

The in vitro specific cell binding of ^64^Cu-anti-hAXL to AXL-expressing human MDA-MB-231 cells was assessed via a competitive method [23]. Briefly, the cells (2 × 10^6^ cells/mL) in binding buffer (PBS with 1% bovine serum albumin [BSA]) were plated in filter plates (96-well; pore size, 0.65 *µ*m; Millipore, Billerica, MA) at 1 × 10^5^ cells/well and incubated at room temperature with ^64^Cu-anti-hAXL (1500 Bq/well) in the presence of increasing concentrations of cold anti-hAXL antibody (0–667 nmol/L, total volume 200 *µ*L) for 2 h. Afterward, the cells were washed three times with PBS (0.1% BSA) and dried, and the radioactivity of the cell-bound filters was measured using a gamma counter. The 50% inhibitory concentration (IC_50_) values for the MDA-MB-231 cells were calculated by fitting the data using GraphPad Prism version 6.05 (GraphPad Software, Inc., La Jolla, CA). Three replicates were conducted for each sample.

### 2.3. Cell Culture

All cell lines were purchased from ATCC (Manassas, VA). Breast carcinoma MCF-7, T47D, and MDA-MB-231 cells were grown in RPMI 1640 medium with 10% fetal bovine serum (FBS), 100 UI/mL penicillin, and 100 *µ*g/mL streptomycin. Hs578T cells were cultured in Dulbecco modified essential medium (DMEM) with 0.01 mg/mL insulin, 10% FBS, 100 UI/mL penicillin, and 100 *µ*g/mL streptomycin. MCF-10A cells were cultured in mammary epithelial growth medium (MCF-10A medium: 1 : 1 Dulbecco's modified Eagle's medium [DMEM]-[F12] medium, 20% horse serum, 0.5 mg/mL hydrocortisone, 10 *µ*g/mL insulin, 20 ng/mL recombinant EGF, 100 ng/mL cholera toxin, and 1 : 100 penicillin/streptomycin [Invitrogen; 15070-063]).

### 2.4. Western Blot Analysis

Whole-cell extracts were subjected to gel electrophoresis and transferred to a polyvinylidene fluoride membrane. Target proteins were probed with goat anti-human AXL primary antibody and Cy-labeled donkey anti-goat secondary antibody (IRDye 800CW). Protein bands were visualized on a fluorescence scanner (Odyssey CLx Infrared Imaging System, LI-COR).

### 2.5. Flow Cytometry Analysis

The cells were collected and stained with human AXL PE-conjugated antibody (FAB154P, R&D Systems) for 30 mins on ice. The PE intensity was measured by BD LSRFortessa Cell Analyzer (BD Biosciences, San Jose, CA). Mouse IgG1 PE-conjugated antibody (IC002P, R&D Systems) was used as isotype-matched control antibody.

### 2.6. Wound-Healing/Scratch Assay

MDA-MB-231 cells were seeded in a 6-well plate (Corning Inc., Corning, NY), 2.5 × 10^5^ cells/well in 2.5 mL RPMI 1640 growth medium. Cells were serum-starved for 24 h when they reached about 90% confluency. The cell monolayer was scratched with a sterile pipet tip to make a “wound.” The growth medium was then removed and the cell layer was washed three times with serum-free medium to remove the detached cells. RPMI 1640 medium (0.5% FBS) plus either 0.5% dimethylsulfoxide (DMSO) or 17-AAG 5 *µ*M was added to each predesigned well, and the cells were incubated as usual. The width of the scratch was documented by microphotograph at 0, 12, 24, and 36 h. The percentage of the wounded area was measured and calculated by using ImageJ version 1.51 (National Institutes of Health, Bethesda, MD).

### 2.7. Real-Time PCR

RNA was extracted from each homogenized tumor using a Direct-zol RNA miniPrep kit (Zymo Research, Irvine, CA) and reverse-transcribed using a Verso cDNA synthesis kit (Thermo Fisher Scientific, Waltham, MA). The reverse-transcribed RNA was subjected to real-time PCR on a Power SYBR PCR master mix (Thermo Fisher Scientific). The following primers were used: vimentin, forward: GACGCCATCAACACCGAGTT, reverse: CTTTGTCGTTGGTTAGCTGGT; Twis1, forward: TCCATTTTCTCCTTCTCTGGAA, reverse: GTCCGCGTCCCACTAGC; Snai1, forward: TCTGAGTGGGTCTGGAGGTG, reverse: CTCTAGGCCCTGGCTGCTAC; Snai2, forward: CAGACCCTGGTTGCTTCAA, reverse: CAGACCCTGGTTGCTTCAA.

### 2.8. Animal Models and MicroPET/CT Imaging

All animal procedures were conducted according to institutional guidelines and were approved by the MD Anderson Cancer Center Institutional Animal Care and Use Committee. Female nude mice, 6 to 8 weeks old, weighing between 20 and 25 g, were purchased from Taconic Biosciences, Inc. (Hudson, NY). Mice were inoculated with MDA-MB-231 cells (5 × 10^6^ in 100 *µ*L Hank balanced salt solution) bilaterally in the mammary fat pads of the lower abdominal wall.

For microPET/CT studies, tumor-bearing mice were injected intravenously with either ^64^Cu-anti-AXL or ^64^Cu-IgG (*n* = 3 mice/group) at a dose of 7.4 MBq/mouse (~0.03 nmol antibody in 200 *µ*L). Twenty-four hours later, they were anesthetized with 2% isoflurane and placed in the prone position. A 15 min PET/CT scan was performed on a Bruker Albira microPET/SPECT/CT scanner (Bruker BioSpin Co., The Woodlands, TX). The system has a resolution of up to 0.7 mm for PET and 90 *µ*m for CT acquisition in all three axes. For data analysis, volumes of interest (VOIs) over the tumors were drawn manually for each scan to record the mean radioactivity. The radioactivity count in each VOI was converted to kBq per cubic centimeter (cc), and the resulting value was divided by the administered dose in MBq and body weight in kg to obtain a standardized uptake value (SUV).

To image the response to HSP90 inhibition, mice were allocated to 2 groups (*n* = 3 mice/group) when the tumor size reached an average diameter of 5 to 7 mm. Each mouse in group 1 received a daily intraperitoneal injection of 17-AAG at a dose of 60 mg/kg/dose in 200 *µ*L volume via a 26-gauge needle for 9 days. Each mouse in group 2 received a daily intraperitoneal injection of vehicle (64% DMSO in PBS, v/v, 200 *µ*L) for 9 days. Tumor dimensions were measured immediately before the first and after the last treatment (day 9) with a Vernier caliper. The mice were injected with ^64^Cu-anti-AXL intravenously on the day of the last 17-AAG or vehicle treatment and subject to imaging 24 h later as already described.

### 2.9. Biodistribution and Autoradiography

All the mice were euthanized immediately after completion of microPET/CT imaging. Major organs and tumors were removed and weighed. The radioactivities of the organ and tumor tissues were counted using a Packard Cobra gamma counter. Organ or tumor uptake of radiotracer was expressed as a percentage of the injected dose per gram of tissue (%ID/g). For autoradiography, the tumor tissues were resected and cryosectioned into 10 *µ*m slices; the slices were exposed on a Fuji image plate (BAS-SR 2025) for 24 h. Each image plate was scanned by a Multifunctional Imaging System (Fuji Film FLA5100, Life Science, Valhalla, NY).

### 2.10. Immunohistochemistry and Western Blot Analyses of Tumor Tissues

Formalin-fixed, paraffin-embedded tumors were processed into 4 *µ*m sections. For immunohistochemistry (IHC) staining, tumor sections were deparaffinized and rehydrated. After antigen retrieval, slides were blocked in PBS with 10% donkey (for hAXL staining) or goat (for vimentin and Ki67 staining) serum and incubated with primary antibodies overnight at 4°C. The antibody dilutions were as follows: hAXL, 1 : 20; vimentin, 1 : 100; Ki67, 1 : 400. The slides were washed and incubated with biotinylated donkey anti-goat and goat anti-rabbit IgG (1 : 200; Vector Laboratories, Burlingame, CA) and streptavidin-conjugated horseradish peroxidase (DAKO, Carpinteria, CA) for 30 min each. The slides were exposed to 3,3′-diaminobenzidine and then counterstained with hematoxylin. The slides were visualized under a Zeiss Axio Observer Z1 microscope (Göttingen, Germany).

For western blot analysis of the tumor tissues, pieces of tumor that had been snap-frozen in liquid nitrogen were homogenized on ice. The lysates were collected after agitation and centrifugation.

### 2.11. Statistical Analysis

Quantitative data are expressed as mean ± standard deviation (SD). Means for each group were compared by using Student's* t*-test.* P* values < 0.05 were considered statistically significant.

## 3. Results

### 3.1. Preparation and Characterization of ^64^Cu-Labeled Anti-hAXL Antibody

As shown in Figure S1 (in Supplementary Material available online at https://doi.org/10.1155/2017/1686525),* p*-SCN-Bn-DOTA was incubated with anti-hAXL or IgG to conjugate the DOTA chelator to the antibody. Anti-hAXL was labeled with ^64^Cu with an efficiency greater than 92% (Figure S2). After purification, the specific radioactivity was 1.50 ± 0.24 MBq/*µ*g for ^64^Cu-anti-hAXL and 2.22 ± 0.29 MBq/*µ*g for ^64^Cu-IgG. After incubation in 20% mouse plasma at 37°C for 2 days, radioactivity associated with the antibodies remained at 90% or more (Figure S3).

### 3.2. AXL Expression on Breast Cancer Cell Lines and Cell Binding Assay

**Figure 1:**
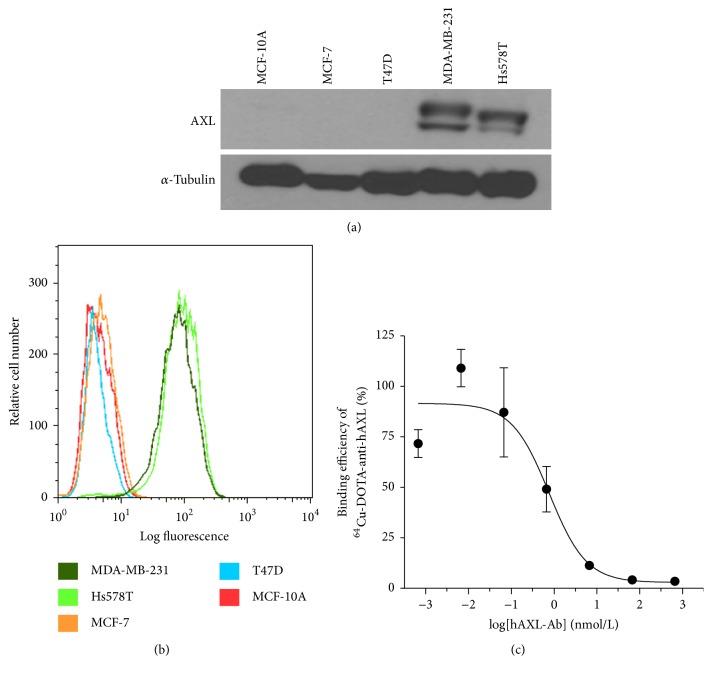
AXL expression of breast cancer cell lines and cell binding assay. (a) Total AXL protein levels in breast cancer cells were determined by western blotting. The two TNBC cell lines, MDA-MB-231 and Hs578T, had high levels of AXL protein expression. (b) Flow cytometry showed high levels of AXL expression on the surface of MDA-MB-231 and Hs578T human TNBC cells. (c) Competitive cell binding assay showed that ^64^Cu-anti-hAXL bound to MDA-MB-231 cells with an IC_50_ value of 0.76 nM.

Expression of AXL was high only in the two TNBC cell lines, MDA-MB-231 and Hs578T, and not in the other two breast cancer cell lines (MCF-7 and T47D) or in the normal human breast epithelial cell line (Figure 1(a)). Flow cytometry data demonstrated the overexpression of AXL on the surfaces of MDA-MB-231 and Hs578T cells (Figure 1(b)). Competitive cell binding experiments showed that the concentration of competing cold anti-hAXL antibody that displaced 50% of the specific binding of ^64^Cu-anti-hAXL (IC_50_) was 0.76 nM (Figure 1(c)).

### 3.3. Pharmacokinetics, In Vivo MicroPET/CT, Autoradiography, and Biodistribution of ^64^Cu-Anti-hAXL in Orthotopic MDA-MB-231 Tumors

**Figure 2:**
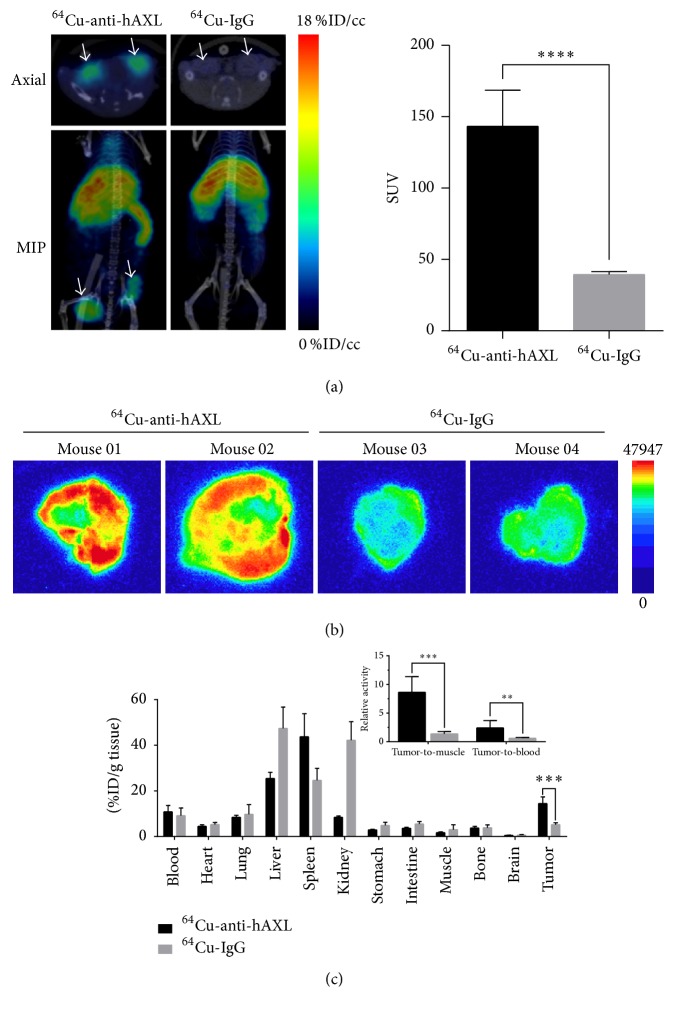
MicroPET/CT imaging of MDA-MB-231 tumors with ^64^Cu-anti-hAXL, autoradiography, and biodistribution. (a) Representative microPET/CT images of MDA-MB-231 tumor xenografts in mice 24 h after intravenous injection of either ^64^Cu-anti-hAXL or ^64^Cu-IgG control. Tumor uptake of ^64^Cu-anti-hAXL, but not ^64^Cu-IgG, was clearly visualized (left). White arrows: tumors. MIP: maximal intensity projection. Quantitative PET analysis of tumor uptake of each radiotracer (*n* = 3/group, right). Tumors of the mice that received ^64^Cu-anti-hAXL had significantly higher uptake of the radiotracer than tumors of the mice that received ^64^Cu-IgG (SUV: 143.20 ± 8.97 versus 39.34 ± 0.85, ^*∗∗∗∗*^*P* < 0.0001). (b) Representative autoradiographs of MDA-MB-231 tumor sections obtained 24 h after intravenous injection of ^64^Cu-anti-hAXL or ^64^Cu-IgG. (c) Biodistribution of radiotracer in mice 24 h after intravenous injection of ^64^Cu-anti-hAXL or ^64^Cu-IgG control (*n* = 3/group). Uptake of ^64^Cu-anti-hAXL in MDA-MB-231 tumors was about three times higher than that of ^64^Cu-IgG (14.48 ± 2.88 versus 5.24 ± 0.76 %ID/g,* P* < 0.001). Inset: comparison of ratios of tumor-to-muscle and tumor-to-blood uptake of ^64^Cu-anti-hAXL and ^64^Cu-IgG (^*∗∗∗*^*P* < 0.001, ^*∗∗*^*P* < 0.01).

The blood activity-time curve for ^64^Cu-anti-hAXL is presented in Figure S4. The half-lives of the radiotracer in the distribution phase (*t*_1/2*α*_) and the elimination phase (*t*_1/2*β*_) were estimated to be 2.15 ± 1.29 h and 47.8 ± 22.5 h, respectively. Therefore, microPET/CT imaging study was performed at 24 h postinjection to better match the blood half-life of the radiotracer and the half-life of the radioisotope (^64^Cu, *t*_1/2_ = 12.7 h). On microPET/CT imaging conducted on tumor-bearing mice 24 h after intravenous injection of ^64^Cu-anti-hAXL or ^64^Cu-IgG control, tumors were delineated by ^64^Cu-anti-hAXL but not ^64^Cu-IgG (Figure 2(a)). Quantitative VOI analyses confirmed that tumor uptake of ^64^Cu-anti-hAXL was significantly greater than that of ^64^Cu-IgG (SUV: 143.20 ± 8.97 versus 39.34 ± 0.85,* P* < 0.0001; Figure 2(a)), a finding that was consistent with the autoradiography results (Figure 2(b)).

After microPET/CT imaging, the imaged mice were subjected to biodistribution studies. The mice were killed immediately and their organs and tumors were collected. The tumor uptake of ^64^Cu-anti-hAXL was nearly three times higher than that of ^64^Cu-IgG (14.48 ± 2.88 versus 5.24 ± 0.76 %ID/g,* P* < 0.001; Figure 2(c)). Similar results were observed in the tumor-to-muscle and tumor-to-blood ratios (Figure 2(c), insert). The tumor-to-muscle ratios were 8.65 ± 2.71 for ^64^Cu-anti-hAXL versus 2.42 ± 1.26 for ^64^Cu-IgG, while the tumor-to-blood ratios were 1.37 ± 0.39 versus 0.60 ± 0.15, respectively.

### 3.4. 17-AAG Downregulated AXL Expression in MDA-MB-231 Cells and Reduced Cell Migration

**Figure 3:**
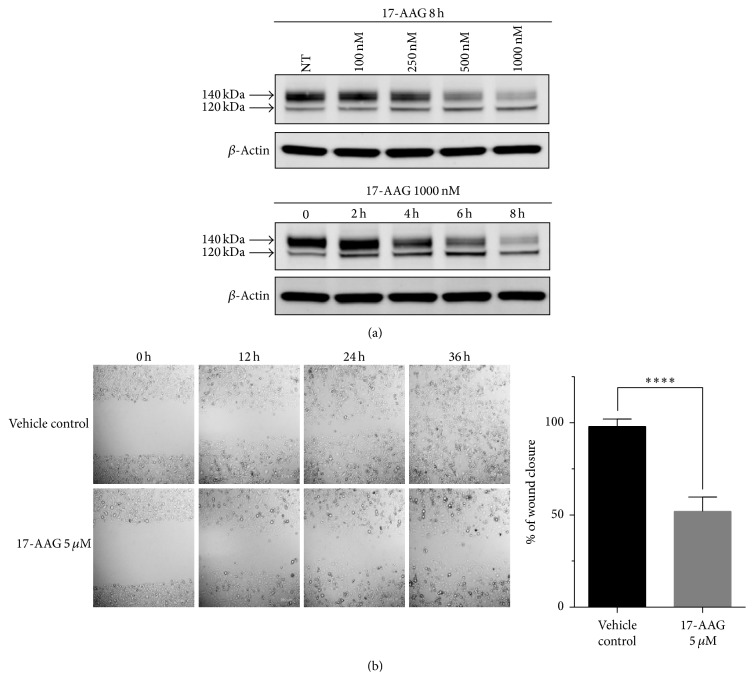
Effects of 17-AAG on AXL expression in and motility of MDA-MB-231 cells. (a) Top panel: protein lysates from MDA-MB-231 cells treated with 17-AAG at the indicated doses for 8 h were immunoblotted for AXL; NT: untreated cells. *β*-Actin was used as a loading control. Bottom panel: protein lysates from MDA-MB-231 cells treated with 1000 nM 17-AAG and harvested at the indicated time points were immunoblotted, again using anti-AXL and anti-actin antibodies. (b) Wound-healing migration assay of MDA-MB-231 cells treated with 17-AAG (5 *µ*M). The healing of the wounds, a measure of cell migration, was imaged at times 0, 12, 24, and 36 h (left). Representative images are shown. Percentage of the closed wound area at 36 h after treatment compared with that at 0 h (right). Results are expressed as means ± SD from three independent experiments (^*∗∗∗∗*^*P* < 0.0001; Student's* t*-test).

The full-length AXL protein has a molecular weight of 104 kDa. Two posttranslationally modified forms of AXL representing products with partial and complete glycosylation (weighing 120 KDa and 140 KDa, resp.) have been identified (http://atlasgeneticsoncology.org). Our western blot analysis showed that 17-AAG primarily decreased the level of the 140-KDa AXL with complete glycosylation in vitro in a dose-dependent manner (Figure 3(a)). Significant downregulation of AXL was observed at a 17-AAG concentration of 0.5 *µ*M and as early as 4 h after treatment (Figure 3(a)). The mechanism of 17-AAG induces AXL degradation and subsequently the inhibition of its oncogenic effects was briefly illustrated in Figure S5.

In the wound-healing scratch assay used to evaluate the effect of 17-AAG on cell migration, a prominent gap persisted at 36 h after scratch in the 17-AAG-treated cells, in contrast to the fully healed scratch in the vehicle-treated cells (*P* < 0.0001; Figure 3(b)).

### 3.5. AXL Downregulation by 17-AAG Was Imaged by MicroPET/CT

**Figure 4:**
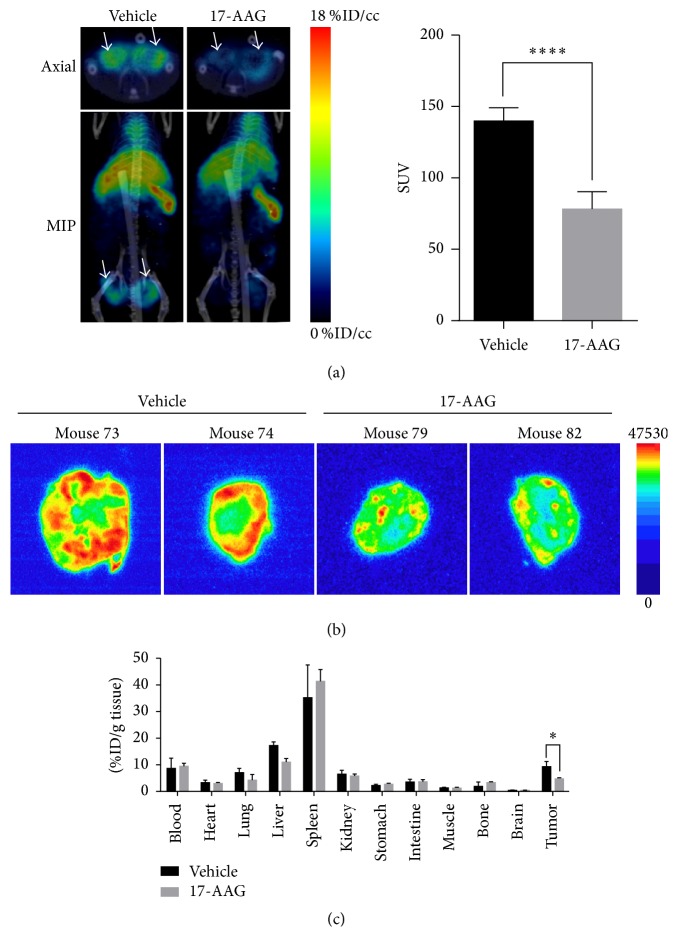
Effect of 17-AAG on AXL in xenograft MDA-MB-231 tumors assessed by microPET/CT imaging and ex vivo autoradiography and radiotracer biodistribution in mice. (a) Representative microPET/CT images of MDA-MB-231 tumor xenografts in mice 24 h after intravenous injection of ^64^Cu-anti-hAXL. One group of mice received a daily intraperitoneal injection of 17-AAG at a dose of 60 mg/kg in 200 *µ*L of DMSO in PBS (128 : 72, v/v) for 9 days before imaging (*n* = 3). Mice in the control group received daily intraperitoneal injections of the same volume of 128 : 72 DMSO/PBS vehicle (*n* = 3) for 9 days before imaging. The tumor uptake of ^64^Cu-anti-hAXL in the 17-AAG treatment group was perceptibly lower than that in the vehicle-treated control group (left). White arrows: tumors. MIP: maximal intensity projection. Quantitative VOI analysis of tumor uptake on microPET/CT images (right). The tumor uptake of ^64^Cu-anti-hAXL was 44% lower in the 17-AAG treatment group than in the vehicle control group (SUV: 78.32 ± 5.02 versus 140.00 ± 3.73, ^*∗∗∗∗*^*P* < 0.0001). (b) Autoradiographs of representative MDA-MB-231 tumor sections from vehicle-treated control mice and 17-AAG-treated mice obtained 24 h after intravenous injection of ^64^Cu-anti-hAXL. (c) Biodistribution of radiotracer 24 h after intravenous injection of ^64^Cu-anti-hAXL in vehicle-treated control mice and 17-AAG–treated mice (*n* = 3/group). The tumor uptake of radiotracer was 49% lower in the 17-AAG treatment group than in the vehicle control group (4.74 ± 1.01 versus 9.31 ± 3.46 %ID/g, ^*∗*^*P* = 0.01).

MicroPET/CT imaging 24 h after injection of ^64^Cu-anti-hAXL detected lower tumor radiotracer uptake in mice that received 17-AAG (once daily for 9 days at 60 mg/kg/dose) than in control mice that received vehicle (*n* = 3/group; Figure 4(a)). Quantitative VOI analysis showed that the tumors of the mice treated with 17-AAG had 44% lower SUV than the tumors of the vehicle-treated mice (78.32 ± 5.02 versus 140.00 ± 3.73,* P* < 0.0001; Figure 4(a)). These data were further confirmed by the autoradiography results, which showed high radioactivity throughout the tumors of vehicle-treated mice but much lower radioactivity in the tumors of the 17-AAG-treated mice (Figure 4(b)). Quantitative analysis by the cut-and-count method showed that the tumor uptake of ^64^Cu-anti-hAXL was 9.31 ± 3.46 %ID/g for the vehicle-treated control mice and 4.74 ± 1.01 %ID/g for the 17-AAG-treated mice (*P* < 0.05; Figure 4(c)).

**Figure 5:**
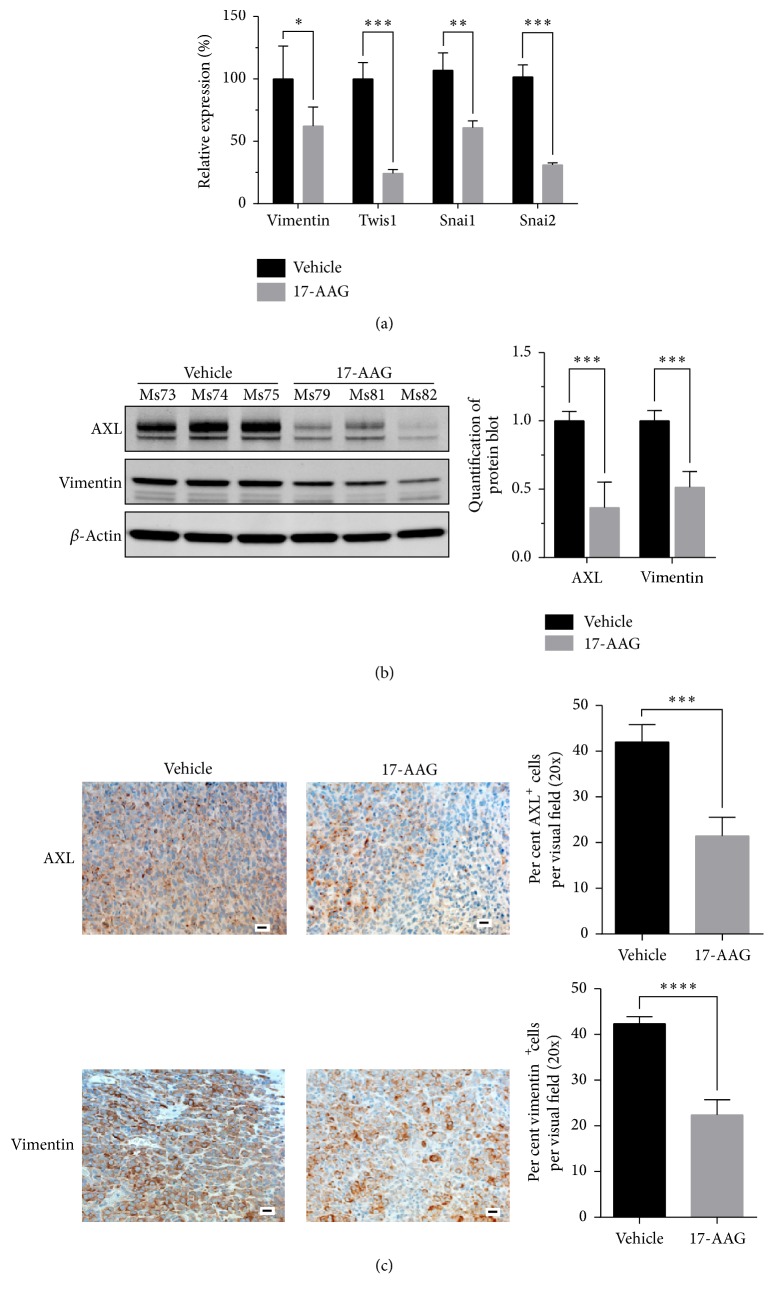
RT-PCR and western blot analysis of tumor lysates and immunohistochemical staining of tumor samples. (a) RT-PCR of MDA-MB-231 tumor lysates shows that tumors from 17-AAG-treated mice had significant lower expression of mRNAs for EMT markers (vimentin, Twis1, Snai1, and Snai2; ^*∗*^*P* < 0.05, ^*∗∗*^*P* < 0.01, ^*∗∗∗*^*P* < 0.001). (b) Left panel: western blot analysis of AXL and vimentin expression in the tumor lysates. Right panel: quantification of protein blots of AXL and vimentin expression in the tumor lysates (^*∗∗∗*^*P* < 0.001). (c) IHC staining of AXL and vimentin expression of the tumor specimens from mice treated with vehicle or 17-AAG. Representative images are shown (scale bar: 20 *µ*m, ^*∗∗∗*^*P* < 0.001, ^*∗∗∗∗*^*P* < 0.0001).

**Figure 6:**
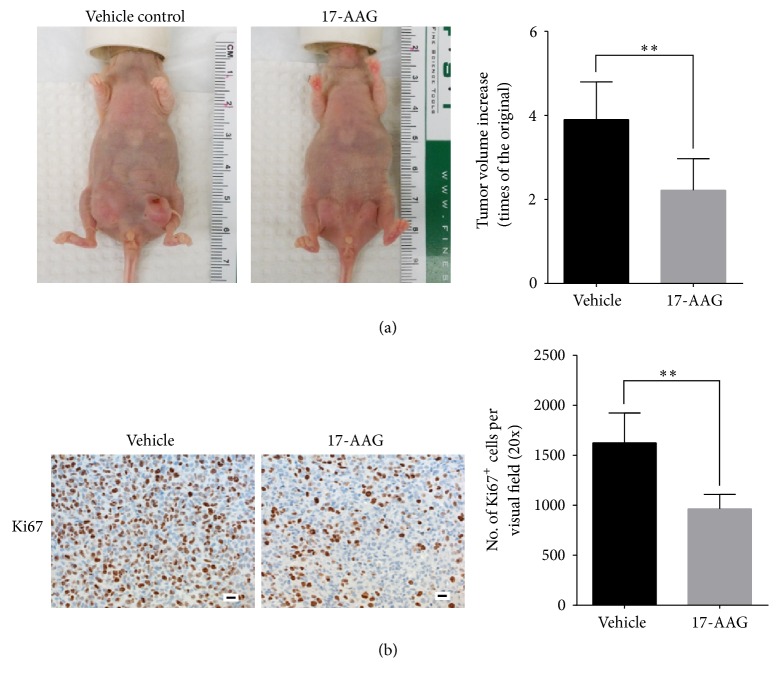
Antitumor effect of Ki67 staining of 17-AAG. (a) Representative IHC staining of Ki67 of tumor specimens from mice treated with vehicle or 17-AAG (scale bar: 20 *µ*m, *n* = 3, ^*∗∗*^*P* < 0.01). (b) Tumor growth delay. The data are expressed as mean fold change after 9 days' treatment in tumor volume ± standard deviation compared to tumor volume measured immediately before initiation of treatment (*n* = 6, ^*∗∗*^*P* < 0.01). Representative photographs of the tumor-bearing mice from the two groups after treatment are shown in the left panel.

We next examined the tumor response to 17-AAG. RT-PCR analysis showed that tumors from mice treated with 17-AAG had significantly lower levels of mRNAs for EMT markers such as vimentin, Twis1, Snai1, and Snai2 than the tumors of control mice (Figure 5(a)). Immunoblotting confirmed the reduction of AXL and vimentin expression in tumors from mice treated with 17-AAG (Figure 5(b)), in accordance with IHC findings (Figure 5(c)). The 17-AAG-treated tumors also exhibited significantly fewer Ki67-positive proliferating cells (Figure 6(a)), which was consistent with delayed tumor growth (2.22 ± 0.34-fold volume increase over tumor volume measure immediately before treatment for the 17-AAG-treated mice versus 3.90 ± 0.37 for the vehicle-treated ones,* P* < 0.01; Figure 6(b)).

## 4. Discussion

In this study, we showed that AXL expression in TNBC MDA-MB-231 tumors can be readily imaged and quantified by using microPET/CT with ^64^Cu-labeled anti-hAXL antibody and that downregulation of AXL in these tumors by HSP90 inhibitor 17-AAG can be noninvasively assessed by microPET/CT imaging.

Noninvasive imaging of TNBC is challenging because these tumors have low expression of imageable biomarkers such as ER and HER-2. Although TNBCs express EGF receptor (EGFR) [5], EGFR inhibitors showed disappointing activity against TNBC in the clinic [24]. We found that AXL was not expressed in normal human breast epithelial MCF-10A cells but was expressed in the two TNBC cell lines of the four breast cancer cell lines we examined. Taken together, these data imply that AXL is a valid therapeutic target in TNBC and that noninvasive assessment of AXL expression in TNBC may be used to predict drug resistance and response to therapies directed at AXL.

Previously, Nimmagadda et al. [25] used single photon emission tomography/CT with ^125^I-labeled anti-AXL antibody to image AXL expression in pancreatic and prostate cancer xenograft tumors. Li et al. [26] used humanized monoclonal anti-AXL antibody (h173) labeled with near-infrared fluorescence dye Cy5.5 to image lung cancer xenografts. Liu et al. [27] used ^64^Cu-labeled h173 to image lung cancer with micro-PET, which showed high blood pool activity and high liver uptake. With *β*^+^ and *β*^−^ emissions and a half-life of about 13 h, ^64^Cu allows for both PET imaging and radionuclide therapy. As a result, ^64^Cu is considered an attractive PET radionuclide for antibody and nanoparticle labeling [28–30]. With a polyclonal anti-human AXL antibody and using* p*-SCN-Bn-DOTA to introduce the radiometal chelator, we achieved both high labeling efficiencies and excellent stability. The labeling stability is especially important, because the dissociation of ^64^Cu from the radiometal chelator would cause accumulation of free ^64^Cu ions in nontarget tissues, which would degrade image quality. Because of the elevated temperature (45°C) and prolonged incubation time (45 min) necessary for high ^64^Cu labeling efficiency, we were concerned whether the AXL binding specificity could be preserved. The fact that ^64^Cu-anti-hAXL bound to AXL-expressing MDA-MB-231 cells could be competitively displaced by cold anti-hAXL with an IC_50_ value of 0.76 nM indicated that ^64^Cu-anti-hAXL specifically bound to MDA-MB-231 cells with high affinity.

The in vivo microPET/CT imaging demonstrated that tumor accumulation of ^64^Cu-anti-hAXL was greater than tumor accumulation of nonspecific ^64^Cu-IgG. In general, the tumor homing of nanoparticles or antibodies can be attributed to both active and passive targeting. Active targeting arises from the specific antigen-antibody interaction in this case, whereas the passive targeting results from the enhanced permeability and retention effect. We included isotype-matched IgG as a control to preclude the passive targeting effect. Both quantitative analysis of microPET/CT images and the cut-and-count measurement showed that the tumor uptake of ^64^Cu-anti-hAXL was significantly higher than that of ^64^Cu-IgG. This was supported by the significantly higher tumor-to-blood and tumor-to-muscle ratios with ^64^Cu-anti-hAXL than with ^64^Cu-IgG. Because of the high cost of commercial anti-hAXL antibody, we did not include cold antibody as the blocking agent for microPET/CT imaging. Nevertheless, differential tumor uptake between ^64^Cu-anti-hAXL and isotype-matched IgG suggests that the former specifically bound to AXL-expressing tumor cells both in vitro and in vivo.

Quantitative analysis showed that uptake of ^64^Cu-anti-hAXL in the tumors of mice treated with 17-AAG was significantly lower than that in the tumors of vehicle-treated mice. Downregulation of AXL in MDA-MB-231 tumors by 17-AAG was confirmed by western blot analysis and IHC. Taken together, these findings show that AXL expression and its pharmacological attenuation could be noninvasively imaged by microPET/CT using ^64^Cu-anti-hAXL as the imaging probe.

A potential application of AXL imaging is based on AXL's role as a client protein of HSP90 [31]. HSP90 is required for proper folding, maturation, and stability of many oncogenic RTKs. Numerous proteins involved in most cellular processes have been identified to be the client proteins of HSP90 chaperoning [32]. The inhibition of HSP90 by 17-AAG depletes ErbB2, EGFR, phosphorylated AKT, and other oncogenic proteins involved in tumor progression. 17-AAG is currently being tested in the clinic [22]. To assess early response and gain pharmacodynamic insights into HSP90 inhibition, radiolabeled antibodies directed against ErbB2 and EGFR have been proposed and tested as potential imageable surrogates to interrogate and quantify the extent of HSP90 inhibition [33, 34]. However, because not all tumors express ErbB2 or EGFR and because HSP90 inhibition can also lead to AXL polyubiquitination and subsequent proteasome degradation [31], noninvasive imaging with ^64^Cu-anti-hAXL may be a useful surrogate for monitoring response to HSP90 inhibition for tumors that do not express ErbB2 or EGFR.

Finally, AXL is known to be an important regulator of EMT. Our findings that treatment with 17-AAG resulted in downregulation of the EMT biomarkers vimentin, Twist, Snai1, and Snai2 at the transcription level and vimentin at the protein level suggest that AXL may be used as a potential biomarker for imaging the pharmacodynamics of EMT inhibition. However, to associate image-derived downregulation of AXL with inhibition of EMT, more tumor models with different levels of AXL expression and their attenuation with different therapeutic approaches should be performed in future studies. Moreover, caution should be taken as ^64^Cu-anti-hAXL showed heterogeneous intratumoral distribution (Figure 4(b)), which may or may not reflect the transcriptional/proteomic responses of EMT pathways of the whole tumors including areas where AXL expression was not detected by ^64^Cu-anti-hAXL.

One limitation of this study is the use of DOTA as the radiometal chelator for ^64^Cu. Although DOTA is still the most widely used chelator, the stability of ^64^Cu-anti-AXL may be further increased with the use of other radiometal chelators such as 1,4,7-triazacyclononane-1,4,7-triacetic acid (NOTA) or cross-bridged cyclam derivatives [35]. Because chelators, linkers, and ligands are all critical to the imaging quality of PET tracers, future efforts should be focused on developing AXL imaging agents with the ultimate goal for translating them into clinical use. Secondly, we used polyclonal antibody in the current work. Further improvement in imaging quality should be made by using engineered monoclonal antibodies with shorter blood half-lives (e.g., single-chain antibody ScFv, diabody, and antibody fragments), or by using a radioisotope with a longer half-life (i.e., ^89^Zr) so that images can be acquired at longer time intervals after injection when radiotracer is largely cleared from normal organs.

## 5. Conclusion

The expression of AXL and its downregulation by 17-AAG could be imaged and quantified by using the ^64^Cu-labeled hAXL. The use of this radioactive probe for noninvasive imaging of AXL, an important target for tumor metastasis and drug resistance, may provide valuable information about dose optimization, dose interval, and therapeutic efficacy for AXL-targeted molecular therapies.

## Supplementary Material

Supplementary Materials include regents and antibodies used, pharmacokinetics, and figures of radiochemistry reaction scheme, radiolabeling efficiency, radiotracer stability, blood activity-time curve, and schematic illustration of the mechanism of 17-AAG induced AXL degradation.

## References

[1] Weigelt B., Peterse J. L., van't Veer L. J. (2005). Breast cancer metastasis: markers and models. *Nature Reviews Cancer*.

[2] Their J. P. (2002). Epithelial-mesenchymal transitions in tumor progression. *Nature Reviews Cancer*.

[3] Thomson S., Petti F., Sujka-Kwok I. (2011). A systems view of epithelial-mesenchymal transition signaling states. *Clinical and Experimental Metastasis*.

[4] Haffty B. G., Yang Q., Reiss M. (2006). Locoregional relapse and distant metastasis in conservatively managed triple negative early-stage breast cancer. *Journal of Clinical Oncology*.

[5] Lehmann B. D., Bauer J. A., Chen X. (2011). Identification of human triple-negative breast cancer subtypes and preclinical models for selection of targeted therapies. *Journal of Clinical Investigation*.

[6] Myers S. H., Brunton V. G., Unciti-Broceta A. (2016). AXL inhibitors in cancer: a medicinal chemistry perspective. *Journal of Medicinal Chemistry*.

[7] Asiedu M. K., Beauchamp-Perez F. D., Ingle J. N., Behrens M. D., Radisky D. C., Knutson K. L. (2014). AXL induces epithelial-to-mesenchymal transition and regulates the function of breast cancer stem cells. *Oncogene*.

[8] Debruyne D. N., Bhatnagar N., Sharma B. (2016). ALK inhibitor resistance in ALKF1174L-driven neuroblastoma is associated with AXL activation and induction of EMT. *Oncogene*.

[9] Wu X., Liu X., Koul S., Lee C. Y., Zhang Z., Halmos B. (2014). AXL kinase as a novel target for cancer therapy. *Oncotarget*.

[10] Shieh Y.-S., Lai C.-Y., Kao Y.-R. (2005). Expression of Axl in lung adenocarcinoma and correlation with tumor progression. *Neoplasia*.

[11] Gjerdrum C., Tiron C., Høiby T. (2010). Axl is an essential epithelial-to-mesenchymal transition-induced regulator of breast cancer metastasis and patient survival. *Proceedings of the National Academy of Sciences of the United States of America*.

[12] Koorstra J. B., Karikari C. A., Feldmann G. (2009). The Axl receptor tyrosine kinase confers an adverse prognostic influence in pancreatic cancer and represents a new therapeutic target. *Cancer Biology & Therapy*.

[13] Brand T. M., Iida M., Stein A. P. (2014). AXL mediates resistance to cetuximab therapy. *Cancer Research*.

[14] Postel-Vinay S., Ashworth A. (2012). AXL and acquired resistance to EGFR inhibitors. *Nature Genetics*.

[15] Meyer A. S., Miller M. A., Gertler F. B., Lauffenburger D. A. (2013). The receptor AXL diversifies EGFR signaling and limits the response to EGFR-targeted inhibitors in triple-negative breast cancer cells. *Science Signaling*.

[16] Bae S. Y., Hong J.-Y., Lee H.-J., Park H. J., Lee S. K. (2015). Targeting the degradation of AXL receptor tyrosine kinase to overcome resistance in gefitinib-resistant non-small cell lung cancer. *Oncotarget*.

[17] Li Y., Ye X., Tan C. (2009). Axl as a potential therapeutic target in cancer: role of Axl in tumor growth, metastasis and angiogenesis. *Oncogene*.

[18] Rankin E. B., Fuh K. C., Taylor T. E. (2010). AXL is an essential factor and therapeutic target for metastatic ovarian cancer. *Cancer Research*.

[19] Sheridan C. (2013). First Axl inhibitor enters clinical trials. *Nature Biotechnology*.

[20] Wu X., Zahari M. S., Ma B. (2015). Global phosphotyrosine survey in triple-negative breast cancer reveals activation of multiple tyrosine kinase signaling pathways. *Oncotarget*.

[21] Jiao Y., Ou W., Meng F., Zhou H., Wang A. (2011). Targeting HSP90 in ovarian cancers with multiple receptor tyrosine kinase coactivation. *Molecular Cancer*.

[22] Khandelwal A., Crowley V. M., Blagg B. S. J. (2016). Natural product inspired N-terminal Hsp90 inhibitors: from bench to bedside?. *Medicinal Research Reviews*.

[23] Wu Y., Zhang X., Xiong Z. (2005). microPET imaging of glioma integrin avb3 expression using (64)Cu-labeled tetrameric RGD peptide. *Journal of Nuclear Medicine*.

[24] Crown J., O'Shaughnessy J., Gullo G. (2012). Emerging targeted therapies in triple-negative breast cancer. *Annals of Oncology*.

[25] Nimmagadda S., Pullambhatla M., Lisok A., Hu C., Maitra A., Pomper M. G. (2014). Imaging Axl expression in pancreatic and prostate cancer xenografts. *Biochemical and Biophysical Research Communications*.

[26] Li D., Liu S., Liu R. (2014). Axl-targeted cancer imaging with humanized antibody h173. *Molecular Imaging and Biology*.

[27] Liu S., Li D., Guo J. (2014). Design, synthesis, and validation of Axl-targeted monoclonal antibody probe for microPET imaging in human lung cancer xenograft. *Molecular Pharmaceutics*.

[28] Anderson C. J., Ferdani R. (2009). Copper-64 radiopharmaceuticals for PET imaging of cancer: advances in preclinical and clinical research. *Cancer Biotherapy and Radiopharmaceuticals*.

[29] Cooper M. S., Ma M. T., Sunassee K. (2012). Comparison of 64cu-complexing bifunctional chelators for radioimmunoconjugation: labeling efficiency, specific activity, and in vitro/in vivo stability. *Bioconjugate Chemistry*.

[30] Zhou M., Zhao J., Tian M. (2015). Radio-photothermal therapy mediated by a single compartment nanoplatform depletes tumor initiating cells and reduces lung metastasis in the orthotopic 4T1 breast tumor model. *Nanoscale*.

[31] Krishnamoorthy G. P., Guida T., Alfano L. (2013). Molecular mechanism of 17-allylamino-17-demethoxygeldanamycin (17-AAG)-induced AXL receptor tyrosine kinase degradation. *Journal of Biological Chemistry*.

[32] Li Y., Zhang T., Schwartz S. J., Sun D. (2009). New developments in Hsp90 inhibitors as anti-cancer therapeutics: Mechanisms, clinical perspective and more potential. *Drug Resistance Updates*.

[33] Smith-Jones P. M., Solit D. B., Akhurst T., Afroze F., Rosen N., Larson S. M. (2004). Imaging the pharmacodynamics of HER2 degradation in response to Hsp90 inhibitors. *Nature Biotechnology*.

[34] Spiegelberg D., Mortensen A. C., Selvaraju R. K., Eriksson O., Stenerlöw B., Nestor M. (2016). Molecular imaging of EGFR and CD44v6 for prediction and response monitoring of HSP90 inhibition in an in vivo squamous cell carcinoma model. *European Journal of Nuclear Medicine and Molecular Imaging*.

[35] Cai Z., Anderson C. J. (2014). Chelators for copper radionuclides in positron emission tomography radiopharmaceuticals. *Journal of Labelled Compounds and Radiopharmaceuticals*.

