# Supplementary material for: MicroPET/CT Imaging of AXL Downregulation by HSP90 Inhibition in Triple-Negative Breast Cancer
Source: Contrast Media Mol Imaging. 2017 May 14;2017:1686525. doi: 10.1155/2017/1686525 (PMC5612679; doi:10.1155/2017/1686525)
Supplement: Supplementary file 1 — Supplementary Materials include regents and antibodies used, pharmacokinetics, and figures of radiochemistry reaction scheme, radiolabeling efficiency, radiotracer stability, blood activity-time curve, and schematic illustration of the mechanism of 17-AAG induced AXL degradation. [file 1686525.f1.docx]

**SUPPORTING INFORMATION**

**MicroPET/CT imaging of AXL downregulation by HSP90 inhibition in triple-negative breast cancer**

Wanqin Wang^1, 3, #^, Jun Zhao^1, #^, Xiaoxia Wen^1^, Curtis Chun-Jen Lin^2^, Junjie Li^1^, Qian Huang^1^, Yongqiang Yu^3,^ *, Shiaw-Yih Lin^2,^ *, and Chun Li^1,^ *

Departments of ^1^Cancer Systems Imaging and ^2^Systems Biology, The University of Texas MD Anderson Cancer Center, Houston, TX, USA; ^3^Department of Radiology, The 1^st^ Affiliated Hospital of Anhui Medical University, Hefei, Anhui province, China.

^#^ Contributed equally to this work.

*Corresponding Authors.

**Regents and Antibodies**

The bifunctional chelating agent S-2-(4-isothiocyanatobenzyl)-1,4,7,10-tetraazacyclododecane-1,4,7,10-tetraacetic acid (*p*-SCN-Bn-DOTA) was purchased from Macrocyclics, Inc. (Dallas, TX). 17-AAG was purchased from Medchem Express (Monmouth Junction, NJ). The goat anti-human polyclonal AXL antibody (AF-154) was purchased from R&D Systems (Minneapolis, MN), and isotype-matched control goat IgG (sc-2028) was purchased from Santa Cruz Biotechnology (Dallas, TX). According to the manufacturer’s brochure, anti-AXL (AF-154) detects human AXL in direct ELISAs and Western blots. The antibody displays less than 25% cross-reactivity with recombinant mouse AXL in ELISA assay (<https://www.rndsystems.com/products/human-AXL-antibody_af154>). The rabbit anti-human monoclonal vimentin antibody (D21H3) and Ki67 antibody (D2H10) were purchased from Cell Signaling Technology, Inc. (Danvers, MA). IRDye 800CW donkey anti-goat secondary antibody was obtained from LI-COR (Lincoln, NE). ^64^CuCl_2_ was supplied by the Cyclotron Radiochemistry Facility at The University of Texas MD Anderson Cancer Center (Houston, TX).

**Pharmacokinetics**

Female nude (8 weeks) received intravenous injection of ^64^Cu-anti-hAXL (4.8 MBq/mouse in 0.2 mL), and blood samples (10 µL) were collected from the tail vein at predetermined time points. Blood half-lives were calculated using classical techniques and the microcomputer-based program Phoenix® WinNonlin® 6.3 software (Pharsight Corp., St. Louis, MO). The blood concentration-time data were best fitted using two compartmental model analysis.

Figure S1. Chemistry and radiochemistry. Schematic illustration of anti-hAXL antibody conjugation with DOTA via amino groups and the benzyl isothiocyanate group of SCN-DOTA. DOTA-anti-hAXL was labeled with ^64^Cu.


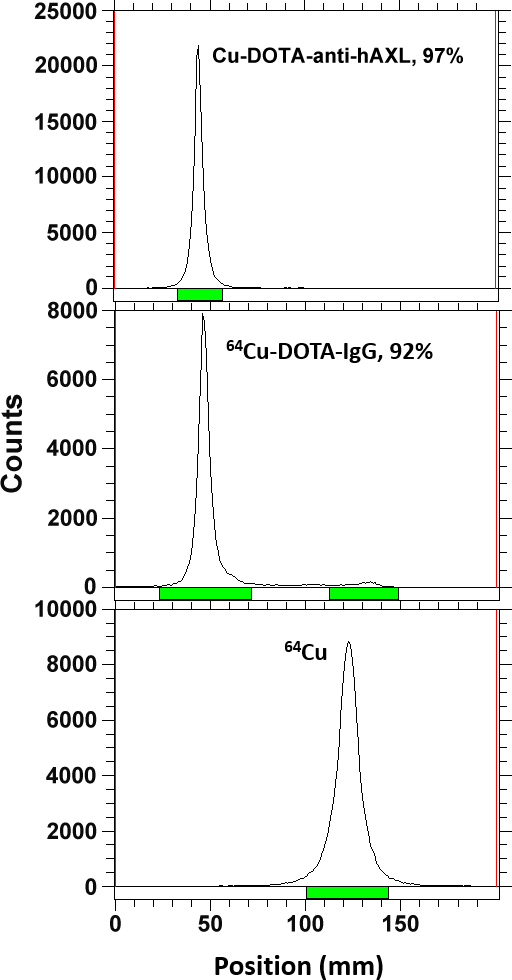


Figure S2. Radiolabeling efficiency of ^64^Cu-DOTA-anti-hAXL and ^64^Cu-DOTA-IgG. Instant thin layer chromatography results show efficiency of ^64^Cu radiolabeling to DOTA-hAXL (upper) and DOTA-IgG (middle); the typical curve and position of ^64^Cu also are shown (lower).


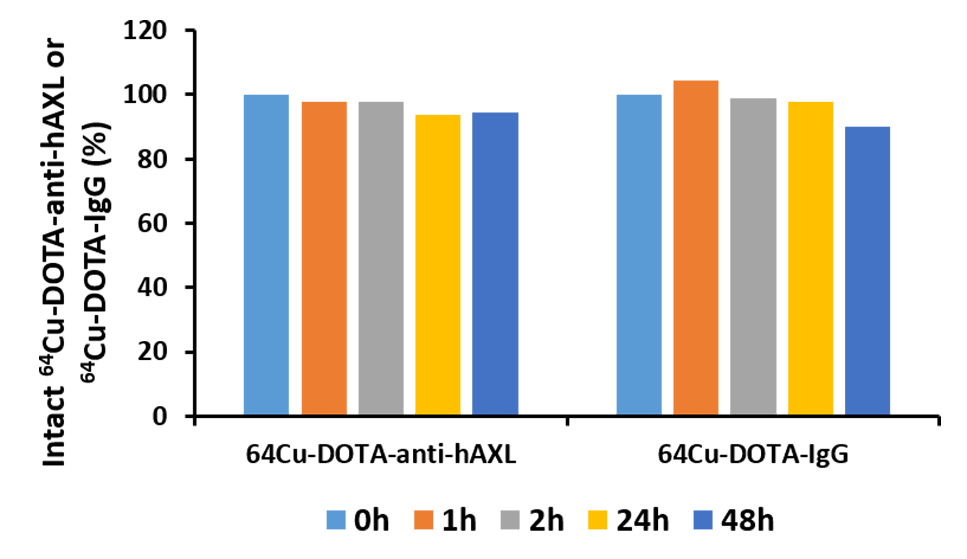


Figure S3. Radiolabeling stability of ^64^Cu-DOTA-anti-hAXL and ^64^Cu-DOTA-IgG. ^64^Cu-labeled antibodies were incubated with 20% mouse plasma at 37°C for 48 h. Radioactivity associated with the antibodies remained at 90% or more.

Figure S4. Blood activity-time curve after intravenous injection of ^64^Cu-anti-hAXL in female nude mice. The data are presented as mean and standard deviation (n = 10).


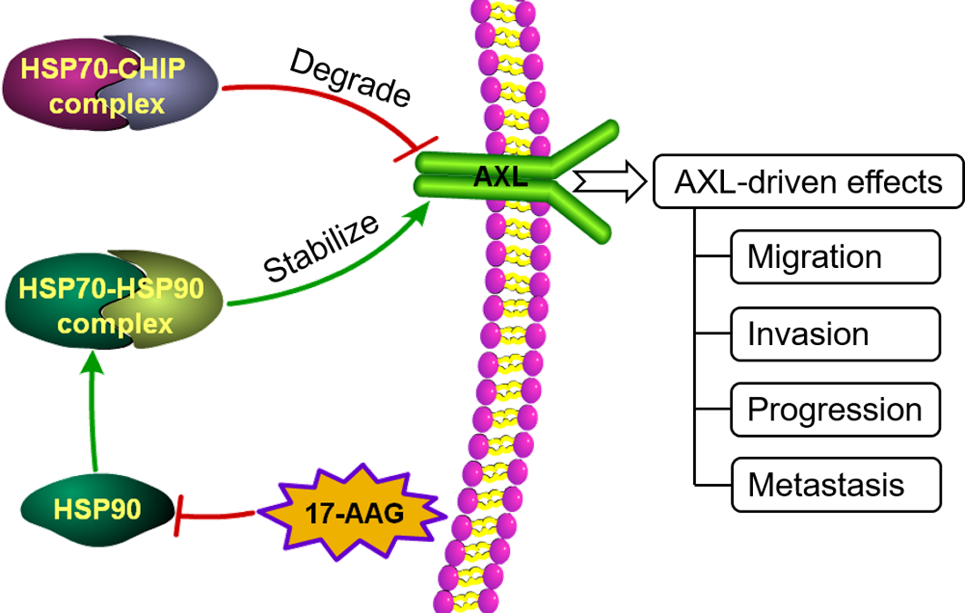


Figure S5. Schematic illustration of the mechanism of 17-AAG–induced AXL degradation and subsequent inhibition of its oncogenic effects.
